# Species-Specific Recognition of *Sulfolobales* Mediated by UV-Inducible Pili and S-Layer Glycosylation Patterns

**DOI:** 10.1128/mBio.03014-19

**Published:** 2020-03-10

**Authors:** Marleen van Wolferen, Asif Shajahan, Kristina Heinrich, Susanne Brenzinger, Ian M. Black, Alexander Wagner, Ariane Briegel, Parastoo Azadi, Sonja-Verena Albers

**Affiliations:** a Molecular Biology of Archaea, Institute of Biology II—Microbiology, University of Freiburg, Freiburg, Germany; b Complex Carbohydrate Research Center, The University of Georgia, Athens, Georgia, USA; c Institute of Biology, Leiden University, Leiden, The Netherlands; d BIOSS Centre for Biological Signaling Studies, University of Freiburg, Freiburg, Germany; University of Vienna

**Keywords:** type IV pili, archaea, *Sulfolobus*, DNA exchange, glycosylation, species-specific recognition

## Abstract

The UV-inducible pili system of *Sulfolobales* (Ups) mediates the formation of species-specific cellular aggregates. Within these aggregates, cells exchange DNA to repair DNA double-strand breaks via homologous recombination. Substitution of the Sulfolobus acidocaldarius pilin subunits UpsA and UpsB with their homologs from Sulfolobus tokodaii showed that these subunits facilitate species-specific aggregation. A region of low conservation within the UpsA homologs is primarily important for this specificity. Aggregation assays in the presence of different sugars showed the importance of *N*-glycosylation in the recognition process. In addition, the *N*-glycan decorating the S-layer of *S. tokodaii* is different from the one of S. acidocaldarius. Therefore, each *Sulfolobus* species seems to have developed a unique UpsA binding pocket and unique *N-*glycan composition to ensure aggregation and, consequently, also DNA exchange with cells from only the same species, which is essential for DNA repair by homologous recombination.

## Abstract (precis)

Type IV pili can be found on the cell surface of many archaea and bacteria where they play important roles in different processes. The UV-inducible pili system of *Sulfolobales* (Ups) pili from the crenarchaeal *Sulfolobales* species are essential in establishing species-specific mating partners, thereby assisting in genome stability. With this work, we show that different *Sulfolobus* species have specific regions in their Ups pili subunits, which allow them to interact only with cells from the same species. Additionally, different *Sulfolobus* species have unique surface-layer *N-*glycosylation patterns. We propose that the unique features of each species allow the recognition of specific mating partners. This knowledge for the first time gives insights into the molecular basis of archaeal self-recognition.

## INTRODUCTION

Type IV pili (T4P) are cell surface appendages that can be found on the cell surfaces of many bacteria and archaea (1, 2). They have been implicated in motility, secretion, DNA transformation, adhesion to surfaces, and the formation of intercellular associations (3, 4). In bacteria, many examples of T4P with cellular binding properties have been described. The major pilin subunit PilE from *Neisseria* T4P was shown to bind endothelial cells and hemagglutinate erythrocytes, whereas the *Neisseria* minor pilin PilV is essential for adherence to host cells (5–10). Additionally, major pilin PilA from Myxococcus xanthus binds to self-produced exopolysaccharides, and subsequent retraction of T4P allows gliding motility and fruiting body formation (11, 12). The major pilin subunit PilA from DNA uptake pili of Vibrio cholerae enables the cells to aggregate specifically with cells from the same species, probably through specific PilA-PilA interactions (13). T4P also form intercellular connections that are essential for conjugational exchange of DNA. For instance, PAPI-1-encoded T4P bring Pseudomonas aeruginosa cells in close proximity by binding to lipopolysaccharides of the recipient cells and, thereby, promote the exchange of PAPI-1 DNA (14, 15).

In archaea, several gene clusters have been found to encode T4P-like structures (4, 16–20). The best-characterized archaeal T4P-like structure is the archaellum, which is essential for swimming motility (4, 20–22). However, little is known about the role and mode of action of archaeal nonarchaellum T4P in attachment to biotic or abiotic surfaces. T4P from the thermophilic crenarchaeon Sulfolobus acidocaldarius (archaeal adhesive pili [Aap]) and the euryarchaea Haloferax volcanii and Methanococcus maripaludis were shown to be involved in attachment to surfaces (23–28). However, their exact mode of binding has not been studied. Next to Aap pili, UV inducible pili of *Sulfolobales* (Ups) pili can be found in *Sulfolobales* (29–32). These T4P assemble upon treatment of the cells with UV stress and other DNA double-strand break-inducing agents. Similar to the above-mentioned T4P of V. cholerae (13), they are crucial in cellular self-interactions, thereby mediating the formation of species-specific cellular aggregates (33, 34). Ups pili thereby provide a mechanism for self-recognition. Within the cellular aggregates, cells are able to exchange chromosomal DNA using the Crenarchaeal exchange of DNA (Ced) system, suggesting a community-based DNA repair system via homologous recombination (33, 35). Interestingly, the Ced system was found to function independently of the Ups pili, even though both systems are essential for DNA transport (36).

The *ups* operon encodes two pilin subunits with a class III signal peptide, namely, UpsA and UpsB (30). Deletion mutants of either *upsA* or *upsB* still form pili (though less and smaller) but do not aggregate after UV induction. The pilins are, therefore, both suggested to be major subunits forming mixed Ups pili (32, 33). While the importance of Ups pili in cellular recognition is known, the underlying molecular mechanism of the species-specific cellular aggregation of *Sulfolobus* species has not been determined.

In this study, we investigated the role of Ups pili in species-specific aggregation on a molecular level. To this end, *in vivo* chimera mutants were constructed in which we exchanged (parts of) the genes encoding the pilin subunits UpsA and UpsB of S. acidocaldarius and Sulfolobus tokodaii. By using these strains in aggregation assays and fluorescence *in situ* hybridization (FISH) experiments, we were able to assign a specific region of UpsA to be required for species-specific cell aggregation of archaeal cells. Furthermore, aggregation assays in the presence of different sugars suggested a role of *N-*glycosylation in cellular recognition. Glycan analysis on the thus far unstudied *S. tokodaii* surface layer (S-layer) showed a different *N-*glycan composition compared to that of other *Sulfolobus* species. Based on these experiments, we propose that a specific region of UpsA forms a binding site to bind species-specific *N-*glycan chains of S-layer components, thereby allowing species-specific cell aggregation and subsequent DNA exchange.

## RESULTS

### The role of pilin subunits in species specificity.

**FIG 1:**
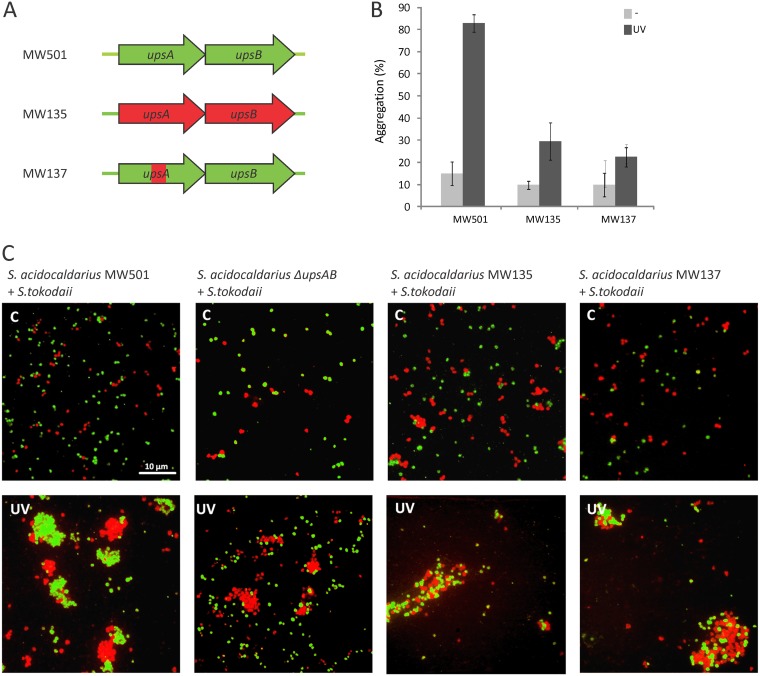
S. acidocaldarius *upsAB* mutants and their aggregation behavior. (A) Schematic overview of genes encoding pilin subunits *upsA* and *upsB* and chimera mutants that were created; (parts of) *upsA* and *B* from S. acidocaldarius (MW501, green) were replaced with the same regions from *S. tokodaii* (red), resulting in MW135 (exchange from start codon of *upsA* until stop codon of *upsB*) and MW137 (exchange of amino acid 84 to 98 in S. acidocaldarius *upsA* with amino acid 80 to 101 of *S. tokodaii upsA*) (see Fig. S2A for an alignment of UpsA from different species). (B) Quantitative analysis of UV-induced cellular aggregation of mutants shown in A. Percentage of cells in aggregates 3 h after induction with or without 75 J/m^2^ UV (dark or light gray, respectively). (C) Aggregation behavior of mixtures of *S. tokodaii* (red) with different S. acidocaldarius mutants (green) after treatment with UV light (UV). Untreated cells were used as a control. Mutants used for this experiment were MW501 (wild-type [WT] *upsAB*), MW143 (Δ*upsAB*), MW135, and MW137. FISH-labeled cells were visualized with fluorescence microscopy. Scale bar, 10 μm.

To study the role of the Ups pilin subunits (UpsA and UpsB) in species-specific recognition of *Sulfolobus* cells, we used S. acidocaldarius MW501 (Δ*flaI*/Δ*aapF*) (Table 1) as a background strain. This strain does not produce archaella or Aap pili (two other type IV pili-like structures present on the cell surface) and was found to aggregate normally upon UV induction (Fig. 1B; see Fig. S1A). The absence of other surface structures enabled unambiguous analysis of Ups pili using electron microscopy. We used our previously established “pop-in pop-out” approach (37) to exchange both *upsA* and *upsB* in this background strain MW501 with the orthologous genetic region from *S. tokodaii* (from the start codon of *upsA* until the stop codon of *upsB*), resulting in strain MW135 (Fig. 1A; Table 1). Upon UV induction, S. acidocaldarius MW135 was still found to produce Ups pili (Fig. S1B); however, interestingly, it showed little to no cellular aggregation (Fig. 1B). To test if this S. acidocaldarius Ups-hybrid strain was able to recognize and, therefore, aggregate with *S. tokodaii* cells, fluorescence *in situ* hybridization with species-specific probes was performed on mixed S. acidocaldarius*/S. tokodaii* strains after UV induction. A positive control with a mixture of background strain S. acidocaldarius MW501 and *S. tokodaii* confirmed previously observed species-specific aggregation (Fig. 1C, first panel). The negative control in which a S. acidocaldarius Δ*upsAB* strain (MW143) was mixed with *S. tokodaii* revealed, as expected, no aggregation of the S. acidocaldarius Δ*upsAB* strain and normal aggregation of *S. tokodaii* (Fig. 1C, second panel). Interestingly, cells from S. acidocaldarius MW135 interacted with *S. tokodaii* cells and, thereby, formed mixed species aggregates (Fig. 1C, third panel). This suggests that the S. acidocaldarius cells expressing *S. tokodaii* Ups pilin subunits were now recognizing and, therefore, interacting with *S. tokodaii* cells.

**TABLE 1:** Strains used and created during this study

Strain	Background strain	Genotype	Source or reference
*S. tokodaii* 7			62
MW001	S. acidocaldarius DSM639	Δ*pyrEF* (91–412 bp)	37
MW501	S. acidocaldarius MW001	Δ*flaI* (Δbp 1–672), Δ*aapF*	32
MW135	S. acidocaldarius MW501	*S. sacidocaldarius upsAB*::*S. tokodaii upsAB*	This study
MW137	S. acidocaldarius MW501	S. acidocaldarius *upsA* (aa^*a*^ 84–98)::*S. tokodaii upsA* (aa 80–101)	This study
MW143	S. acidocaldarius MW501	Δ*upsAB*	This study

a aa, amino acid.

10.1128/mBio.03014-19.2 FIG S1 (A) UV-induced cellular aggregation of S. acidocaldarius MW501. Representative differential inference contrast (DIC) microscopy images of noninduced cells (C) and cells 3 hours after UV induction (UV) with 75 J/m^2^ UV. Scale bar, 5 μm. (B) Transmission electron micrographs of UV-induced S. acidocaldarius mutants (top panel) and expression strains (bottom panel). Top panel: Ups pili of S. acidocaldarius MW501 (Δ*flaI*/Δ*aapF*), MW135 (exchange S. acidocaldarius *upsAB* genes with those of *S. tokodaii* from start codon of *upsA* until stop codon of *upsB*), and MW137 (*Saci upsA* aa 84 to 98::*ST upsA* aa 80 to 101). Bottom panel: UV-induced S. acidocaldarius expression strains. Wild-type and mutated *upsAB* genes (black squares in Fig. S2A) were expressed in a Δ*upsAB/*Δ*flaI*/Δ*aapF* strain (MW143). The following maltose-inducible expression plasmids were used: pSVA1855 expressing wild-type *upsAB*; pSVA1860, expressing *upsAB* with a D85A mutation in *upsA*; pSVA1860, expressing *upsAB* with a D85A mutation in *upsA*; pSVA1861, expressing *upsAB* with a N87A mutation in *upsA*; pSVA1862, expressing *upsAB* with a N94A mutation in *upsA*; and pSVA1863, expressing *upsAB* with a Y96A mutation in *upsA.* Scale bar, 100 nm. Download FIG S1, TIF file, 2.3 MB. Copyright © 2020 van Wolferen et al. 2020 van Wolferen et al. This content is distributed under the terms of the Creative Commons Attribution 4.0 International license.

10.1128/mBio.03014-19.3 FIG S2 (A) Alignments of UpsA from different *Sulfolobales*. The class III cleavage site, cleaved by PibD, is depicted by a red line. The red box in UpsA indicates the less conserved region. Shown are UpsA amino acid sequences from Sulfolobus acidocaldarius DSM 639 (*Saci*), Sulfolobus tokodaii strain 7 (*ST*), Sulfolobus solfataricus P2 (*Sso*), Stygiolobus azoricus (*Staz*), Metallosphaera cuprina Ar-4 (*Mcup*), Metallosphaera hakonensis DSM 7519 (*Mhak*), Metallosphaera sedula DSM5348 (*Msed*), and Metallosphaera yellowstonensis MK1 (*Myel*). (B) Maximum-likelihood phylogenetic tree of UpsA and UpsB homologs from different archaeal species (Sulfolobus acidocaldarius strains DSM 639, N8, Ron12/I, and SUSAZ; Sulfolobus solfataricus strains P2 and 98/2, P1; Sulfolobus islandicus strains REY15A, HVE10/4, and M.16.4; Sulfolobus tokodaii strain 7; Stygiolobus azoricus; Metallosphaera sedula DSM5348; Metallosphaera cuprina Ar-4; Metallosphaera hakonensis DSM 7519; and *Metallosphaera yellowstonensis* MK1). Branch numbers represent bootstrap values above 80% (100 replicates). Download FIG S2, TIF file, 2.8 MB. Copyright © 2020 van Wolferen et al. 2020 van Wolferen et al. This content is distributed under the terms of the Creative Commons Attribution 4.0 International license.

To find putative species-specific regions in the pilin subunits involved in species-specific recognition, alignments were made using UpsA and UpsB amino acid sequences, from several *Sulfolobales* (see Fig. S2A). Additionally, the relationship between UpsA and UpsB homologs was studied by creating a phylogenetic tree (Fig. S2B, Text S1). A region with low conservation was revealed in UpsA (Fig. S2A, amino acid 84 to 98 for S. acidocaldarius, red box). To test whether this region plays a role in cell-cell recognition, the region of low conservation in S. acidocaldarius UpsA (amino acid 84 to 98) was exchanged with the corresponding part from *S. tokodaii* UpsA (amino acid 80 to 101) (resulting in strain MW137) (Fig. 1A; Table 1). Similar to what was observed for the S. acidocaldarius mutant in which *upsA* and *upsB* were exchanged completely (MW135), S. acidocaldarius MW137 still formed Ups pili (Fig. S1B) but showed little to no UV-inducible aggregation with itself (Fig. 1B). Instead, it was found to aggregate with *S. tokodaii* (Fig. 1C, fourth panel). This observation strongly suggests that the nonconserved region (exchanged in MW137) defines the species specificity during cellular aggregation.

10.1128/mBio.03014-19.1 TEXT S1 Describing the phylogenetic analysis of UpsA and UpsB from different *Sulfolobales* and the *N-*glycan analysis of the glycosylated S-layer of *S. tokodaii* S-layer proteins SlaA and SlaB. Download Text S1, DOCX file, 0.03 MB. Copyright © 2020 van Wolferen et al. 2020 van Wolferen et al. This content is distributed under the terms of the Creative Commons Attribution 4.0 International license.

### The role of glycosylation in species specificity.

**FIG 2:**
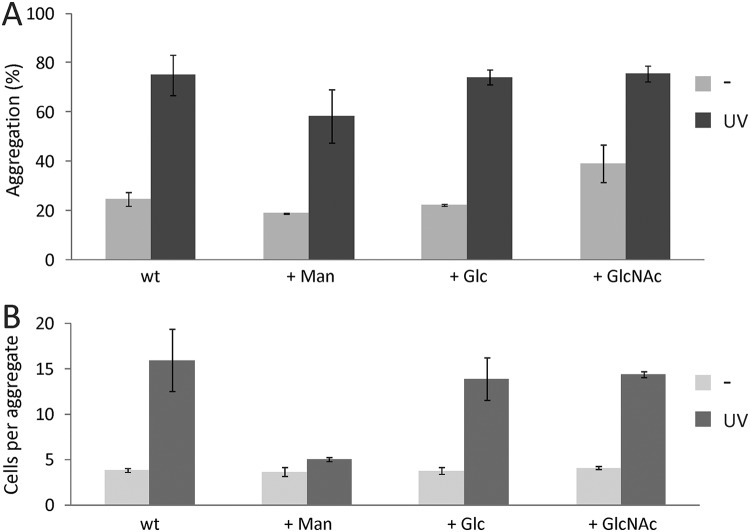
UV-induced aggregation of S. acidocaldarius MW001 upon addition of 20 mM mannose, glucose, or *N*-acetylglucosamin*e*. (A) Percentage of cells in aggregates. (B) Average sizes of formed aggregates. Light gray bars represent noninduced cells, and dark gray bars represent cells induced with 75 J/m^2^ UV.

The fact that *Sulfolobus* Ups wild-type strains are able to form mating pairs with Ups-deletion strains (33) suggests that factors other than Ups pili play a role in species-specific recognition. All *Sulfolobales* harbor an S-layer containing two proteins, namely, SlaA and SlaB. Both proteins are heavily glycosylated, and the cells are thereby fully covered in an extensive extracellular glycan layer (38–40). We, therefore, suggested that Ups pili might recognize glycosylated proteins and, thereby, initiate cellular interactions. To confirm this hypothesis, UV-induced aggregation assays were performed in the presence of monosaccharides that are also part of the S. acidocaldarius *N*-glycan chain (Glc_1_Man_2_GlcNAc_2_QuiS, containing glucose, mannose, *N-acetylglucosamine*, and the *Sulfolobus*-specific sulfoquinovose residues) (39) (Fig. 2). The addition of *N*-acetylglucosamine or glucose did not result in altered cellular aggregation (Fig. 2A and B); however, in the presence of mannose, cell aggregates were significantly smaller (Fig. 2B). To verify that the observed reduced aggregation was not caused by a lower expression of the pilin genes, we performed quantitative PCR (qPCR) on cDNA from cells isolated after the addition of mannose. We could not observe any differences in *upsA* transcript levels between cells that were or were not incubated with mannose, independent of UV treatment (see Fig. S3). Thus, we assume that Ups pili expression is not affected by the addition of mannose. Our results, therefore, suggest that mannose molecules partially saturate the binding sites of the Ups pili and, thereby, inhibit interactions between pili and the glycan chains on the S-layer of the host cell, resulting in reduced aggregation.

10.1128/mBio.03014-19.4 FIG S3 Effect of mannose on transcript levels of *upsA* genes in S. acidocaldarius MW001 after treatment with UV (3 hours after treatment with 75 J/m^2^ UV) (right) or not (left) as measured by reverse transcription-quantitative PCR (qRT-PCR). Differences are displayed as log_2_ fold changes. Used primers are summarized in Table S1. Download FIG S3, TIF file, 0.9 MB. Copyright © 2020 van Wolferen et al. 2020 van Wolferen et al. This content is distributed under the terms of the Creative Commons Attribution 4.0 International license.

### Defining the glycosylation pattern of *S. tokodaii* S-layer proteins.

**FIG 3:**
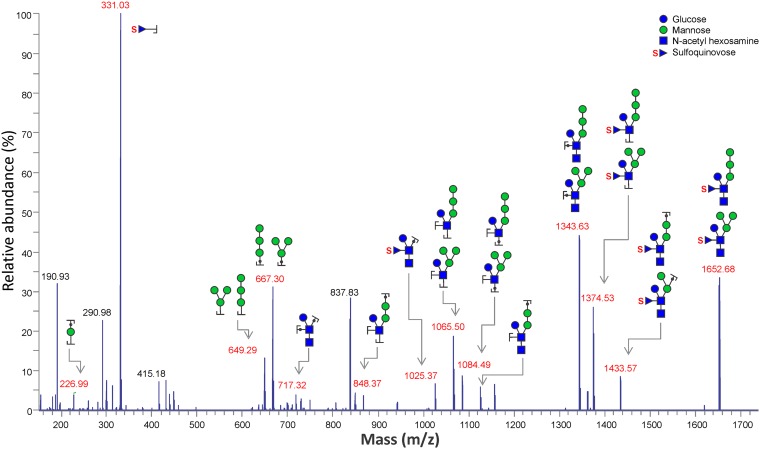
High cell density (HCD) MS^2^ spectra of heptasaccharide (*m/z*, 1,651.7) (Fig. S4a) released from the S-layer proteins from *S. tokodaii* by hydrazinolysis.

**FIG 4:**
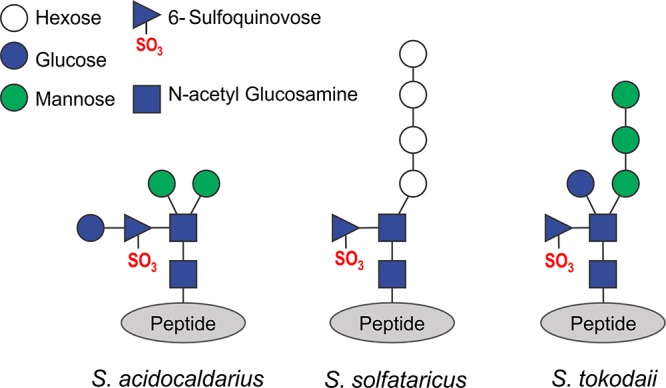
Structure of the glycan trees present on the S-layer of *S. tokodaii* compared with those from S. acidocaldarius (39) and S. solfataricus (42).

Our hypothesis that S-layer glycosylation is important for species specificity suggests that different *Sulfolobus* species have different glycosylation patterns. So far, the glycan structure of *S. tokodaii* is unknown. To analyze the glycan structures on the S-layer of *S. tokodaii*, *N*-glycans were released from isolated S-layer by hydrazinolysis. Using matrix-assisted laser desorption ionization–time of flight mass spectrometry (MALDI-TOF-MS) profiling, one main *N*-glycan species and two other low-abundant species could be identified in both positive (see Fig. S4A) and negative ion mode (Fig. S4B). The structures of *N-*glycans were proposed based on mass-to-charge ratio of each *N*-glycan ions observed (Fig. S4; Table 2) as well as its tandem mass spectrometry (MS^2^) fragmentation pattern (Fig. 3). The three *N-*glycan species were identified as QuiS_1_Hex_4_HexNAc_2_, QuiS_1_Hex_3_HexNAc_2_, and QuiS_1_Hex_4_HexNAc_1_ (Table 2). To determine the linkages between the sugars in the deduced *N*-glycan species, linkage analysis (41) was performed on the permethylated *N-*glycans released from S-layer proteins. The various types of linkages observed on each monosaccharide and their relative abundances on the *N-*glycans are shown in Fig. S5. The most plausible position of this linkage in the glycan chain can be observed on the right-side column in Fig. S5. Based on this linkage information, sequential mass spectrometry (MS^n^) determination of glycan branching (Fig. 3) and the glycan masses (Fig. S4), the *N*-glycan glycoforms, and their isomers were deduced (see Fig. S6). Fig. 4 schematically shows the most prominent glycan structures from S. acidocaldarius (39), Sulfolobus solfataricus (42), and *S. tokodaii* (this study). In agreement with our hypothesis, the core of these structures is similar, whereas the terminal saccharides differ. A typical sulfated sugar residue is present in all three *Sulfolobus* glycan structures. Using liquid chromatography (LC)-MS profiling on the tryptic digest of S-layer proteins SlaA and SlaB, several different glycopeptides could indeed be observed (Text S1, Fig. S7 and S8, respectively).

**TABLE 2:** List of *N*-linked glycans released from S-layer glycoprotein from *S. tokodaii* detected by MALDI-TOF-MS^*a*^

Permethylated mass (*m/z*) by mode	Text description of structures	% of glycans^*d*^
Positive ion^*b*^		
1,406	QuiS _1_Hex_4_HexNAc_1_	3.92
1,447	QuiS _1_Hex_3_HexNAc_2_	7.09
1,651	QuiS_1_Hex_4_HexNAc_2_	88.99
Negative ion mode^*c*^		
1,360	QuiS _1_Hex_4_HexNAc_1_	1.27
1,401	QuiS _1_Hex_3_HexNAc_2_	1.82
1,605	QuiS _1_Hex_4_HexNAc_2_	96.91

a QuiS, sulfoquinovose; Hex, hexose; HexNAc, *N-*acetyl hexosamine.

b All masses (mass + 2Na − H) are single charged.

c All masses (mass − H) are single charged.

d Calculated from the area units of detected *N*-linked glycans.

10.1128/mBio.03014-19.5 FIG S4 (A) MALDI MS spectra of *N*-glycans released from the S-layer protein from *S. tokodaii* by hydrazinolysis observed (positive ion mode). (B) MALDI MS spectra of *N*-glycans released from the S-layer protein from *S. tokodaii* by hydrazinolysis observed (negative ion mode). Structures are assigned based on MS^2^ analysis.*, During hydrazinolysis, a fraction of glycans gets derivatized by hydrazine reagent. Download FIG S4, TIF file, 0.7 MB. Copyright © 2020 van Wolferen et al. 2020 van Wolferen et al. This content is distributed under the terms of the Creative Commons Attribution 4.0 International license.

10.1128/mBio.03014-19.6 FIG S5 Glycosyl linkages of monosaccharides of *N*-glycans from the S-layer proteins of *S. tokodaii* were determined by gas chromatography (GC)-MS analysis using the partially methylated alditol acetate (PMAA) method. Download FIG S5, TIF file, 1.8 MB. Copyright © 2020 van Wolferen et al. 2020 van Wolferen et al. This content is distributed under the terms of the Creative Commons Attribution 4.0 International license.

10.1128/mBio.03014-19.7 FIG S6 Different possible glycoforms of *N*-glycans identified on the S-layer proteins from *S. tokodaii*. Multiple isomers of each glycoforms were also observed. The structure, branching, and linkage of *N-*glycans were characterized by MS^n^ fragmentation by electrospray ionization (ESI)-MS^n^ and linkage analysis by GC-MS. Download FIG S6, TIF file, 1.0 MB. Copyright © 2020 van Wolferen et al. 2020 van Wolferen et al. This content is distributed under the terms of the Creative Commons Attribution 4.0 International license.

10.1128/mBio.03014-19.8 FIG S7 *N-*linked glycosylation sites identified from SlaA of *S. tokodaii* by LC-MS^2^ analysis (tryptic digestion and semispecific cleavage search using Byonic software). (A) HCD MS^2^ spectra of glycopeptide ^1175^IYYN[SuphoQuinovose_1_Hex_4_HexNAc_2_] ATSGR^1183^ from SlaA. (B) HCD MS^2^ spectra of glycopeptide ^1188^NVYGQVVLN[SuphoQuinovose_1_Hex_4_HexNAc_2_]AS GN^1200^ from SlaA. (C) HCD MS^2^ spectra of glycopeptide ^1222^AVLPN[SuphoQuinovose_1_Hex_4_HexNAc_2_]NTLTTL TFNK^1236^ from SlaA. (D) HCD MS^2^ spectra of glycopeptide ^1298^IIPAN[SuphoQuinovose_1_Hex_4_HexNAc_2_]ITPIR^1307^ from SlaA. (E) HCD MS^2^ spectra of glycopeptide ^1362^EGVN[SuphoQuinovose_1_Hex_4_HexNAc_2_]ASVTSPV VYYSYQAV VAK^1383^ from SlaA. (F) HCD MS^2^ spectra of glycopeptide ^1421^AVGPAISEYPVNLVFTN[SuphoQuinovose_1_Hex_4_ HexNAc_2_]VT VEK^1442^ from SlaA. Download FIG S7, TIF file, 1.9 MB. Copyright © 2020 van Wolferen et al. 2020 van Wolferen et al. This content is distributed under the terms of the Creative Commons Attribution 4.0 International license.

### Determination of the binding site in UpsA.

**FIG 5:**
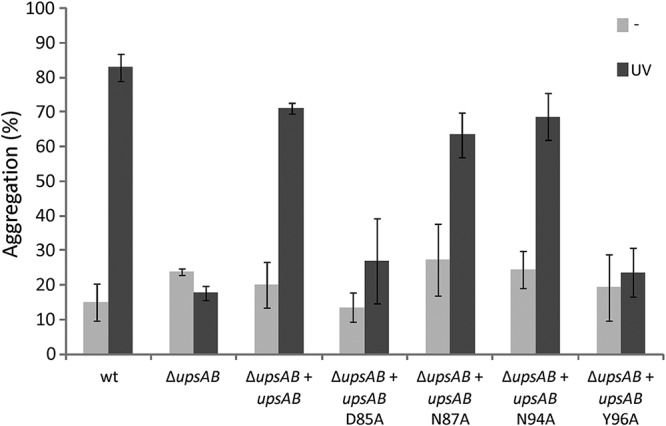
UV-induced cellular aggregation of S. acidocaldarius Δ*upsAB* complementation strains. A S. acidocaldarius Δ*upsAB* mutant (MW143) was complemented with maltose-inducible plasmids carrying *upsAB* or *upsAB* with a D85A, N87, N94A, or Y96A mutation in UpsA (see also Fig. S2A). Percentage of cells in aggregates 3 h after induction with or without 75 J/m^2^ UV (dark or light gray, respectively).

We know that a S. acidocaldarius mutant in which both Ups pilin subunits are deleted does not aggregate upon UV stress (32). Here, we could successfully complement this phenotype by expressing the *upsAB* genes from a maltose-inducible plasmid (Fig. 5) (Δ*upsAB* + *upsAB*). Using site-directed mutagenesis on this plasmid, we, moreover, created point mutations within the above-described region of interest of UpsA (black squares in Fig. S2A), namely, D85A, N87A, N94A, and Y96A. All mutants still produced Ups pili upon UV induction (Fig. S1B). Interestingly, when expressing UpsA in which the poorly conserved residues D85 or Y96 were mutated to alanine, UV-induced aggregation was significantly reduced. On the other hand, mutation of conserved N87 or N94 showed wild-type aggregation (Fig. 5). These results suggest that the region of low conservation within UpsA is specifically adapted to the glycan structure of the same species to ensure species-specific aggregation.

## DISCUSSION

Both bacterial and archaeal T4P have shown to be essential for surface adherence. Given the fact that bacterial T4P are strongly related to pathogenicity, their mode of binding has primarily been studied for pathogenic bacteria, such as P. aeruginosa, V. cholerae, *Neisseria*, and enteropathogenic Escherichia coli species. However, nonpathogenic bacteria and archaea also carry several T4P involved in adhesion, which are studied in far less detail. The crenarchaeal *Sulfolobales* carry the following three types of T4P: archaella, involved in swimming motility (21); Aap pili, involved in attachment to diverse surfaces (25, 26); and Ups pili, mediating intraspecies cellular aggregation and DNA exchange (30, 32, 33, 36). During this study, we have examined the role that Ups pili play in the formation of *Sulfolobus* mating partners. In particular, we focused on the role that pilin subunit UpsA plays in cell recognition.

The Ups pilus is formed by two pilin subunits, UpsA and UpsB, which are both thought to be major pilin subunits that build up mixed pili structures (32). We revealed that UpsA is involved in species-specific cellular interactions, and we were able to alter this specificity by exchanging (parts of) the pilin subunit with that of another species (Fig. 1). The binding of bacterial surface structures to other cells is often based on interactions with sugars (43, 44). Surface-exposed glycans can be found on cells from all domains of life where they display an enormous range of different structures that are often highly specific to certain species (45). Glycans are, therefore, perfect anchors to bind specific host or partner cells.

**FIG 6:**
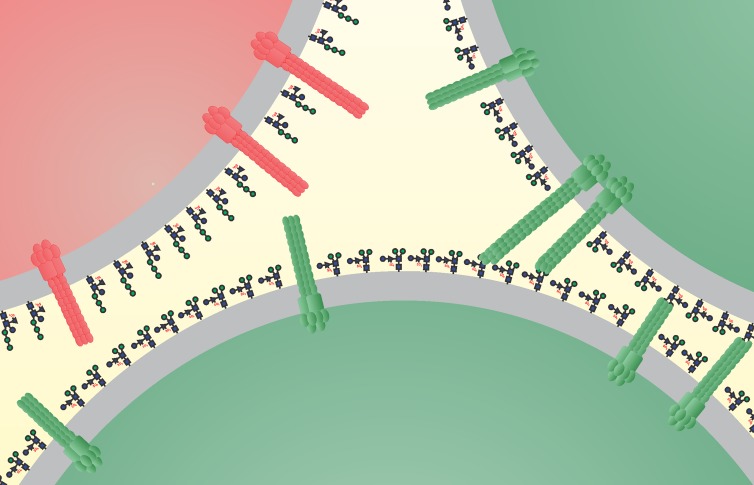
Proposed model of species-specific interactions between Ups pili and *N-*glycosylated S-layer of *Sulfolobales*. Ups pili of S. acidocaldarius (green) only form interactions with the *N-*glycan of the same species and not with that of other species (*S. tokodaii*, red).

The glycosylation ratio of S-layer protein SlaA from S. acidocaldarius was found to be extremely high compared with that of S-layer proteins from euryarchaeal species. This high glycosylation density is thought to be an adaptation to the high temperature and acidic environment that *Sulfolobus* species live in (39). Recently, the extensive lawn of glycans on top of the S-layer of S. solfataricus was visualized by cryo-electron microscopy (EM), emphasizing the general importance of *Sulfolobus N-*glycosylation in the formation of cellular interactions with anything that is present in the extracellular environment (40). In Saccharomyces cerevisiae, surface-exposed lectins can bind to surface-exposed sugars in a calcium-dependent manner, thereby forming cellular aggregates, a process which is called flocculation (46). This behavior can be inhibited by saturating the binding of the lectins through the addition of loose sugars to the medium (47) (Fig. 2). In similar experiments with S. acidocaldarius, we found that mannose has an inhibiting effect on UV-induced cellular aggregation. Since two outer mannose residues are present in the S. acidocaldarius *N*-glycan tree, binding of Ups pili to this side of the glycan tree is probable. When analyzing the *N-*glycans of *S. tokodaii*, we could indeed find differences in the outer part of the *N*-glycan structure compared with that of S. acidocaldarius (39) and S. solfataricus. (42) (Fig. 4). As observed for *Eukarya* (48), the core or the glycan structure is similar in all three species, whereas the outer residues differ. Our results, thereby, suggest that UpsA contains a specific binding pocket that is able to bind specific sugar moieties of the *N-*glycans presented on the S-layer of distinct *Sulfolobus* species (Fig. 6).

Among the euryarchaeal *Haloferax* species, glycosylation was found to be essential for cell fusion (49), emphasizing the importance of glycosylation in archaeal cellular recognition in general. It is unclear if pili or other types of lectin molecules are involved in cellular interactions that initiate *Haloferax* fusion events. Similar to our findings, different *Haloferax* species are also known to be differentially glycosylated (50), leading to semispecific cell-cell recognition (49). Cell fusion between different *Haloferax* species could also be observed but with far lower efficiency (51). In addition, under different environmental conditions, *Haloferax* glycosylation patterns change, leading to more or less favorable *N-*glycans for mating (52). One could envision that low-frequency interactions between different *Sulfolobus* species also occur and might occasionally lead to horizontal gene transfer (35), thereby playing an important role in genome evolution. In a single hot spring in Kamchatka, Russia, two different groups of Sulfolobus islandicus strains were found to be present. Despite their coexistence, it was postulated that S. islandicus species mainly exchange DNA within these groups (53). It is likely that *N*-glycan patterns and Ups pili between the species are different, serving as a barrier to gene transfer. This behavior might be seen as the two groups diverging into different species. Bacterial T4P are dynamic structures that can polymerize and depolymerize, allowing cycles of pili extension and retraction and enabling cells to pull themselves toward other (host) cells and surfaces (1). So far, nothing is known about the dynamics of Ups pili, and it is unclear how interactions between Ups pili and glycosylated S-layer result in the formation of mating pairs. We assume that for the cells to exchange DNA, tight cellular interactions have to be formed that enable direct cell surface contact. If cellular interactions between *Sulfolobus* cells are initiated with the tip of an Ups pilus, the distance created by the Ups pili themselves will have to be overcome. Unlike bacteria, archaea do not carry homologs of the PilT ATPase allowing retraction of T4P (54). Recently, pili retraction has been observed in certain bacterial T4P systems that lack a retraction ATPase (55, 56); in addition, retraction could be observed in certain PilT deletion mutants (although with far lower force) (57–59). One could, therefore, imagine that PilT-independent retraction by multiple Ups pili will create a collective force that is strong enough to pull cells together and form tight aggregates. It can, of course, not be excluded that so far unidentified retraction ATPases are involved in retraction of Ups pili. Alternatively, one could imagine scenarios in which Ups pili are degraded extracellularly and, thereby, shortened until cell-cell contact is established, or they could be flexible enough to completely bend toward the cell surface.

The Ced system that is involved in DNA transfer among *Sulfolobales* can also be found in several crenarchaea that do not encode Ups pili (35, 36); it is, therefore, likely these species have developed a different mechanism to initiate cellular interactions. Given the importance of glycosylation in cell-cell interactions in both euryarchaeal *Haloferax* and crenarchaeal *Sulfolobus* species, glycosylation is likely to play a role in these interactions.

This study has given molecular insights in the cellular recognition mechanism of the described crenarchaeal Ups system (30, 32, 33). Our current model suggests that upon DNA damage, Ups pili are formed; the UpsA pilin subunits contain a species-specific glycan-binding pocket in pilin subunit UpsA that can bind glycans presented on cells from the same species (Fig. 6). This system allows the formation of species-specific cellular connections prior to DNA exchange via the Ced system (36). In that way, only DNA from the same species is exchanged and used for DNA repair via efficient homologous recombination. This proposed cellular recognition mechanism in *Sulfolobales* promotes the exchange of genomic DNA to cells from the same species, thereby playing an important in role genome integrity and the maintenance of species. Coevolution of *N-*glycosylation and pilin subunit UpsA might play an important role in speciation.

## MATERIALS AND METHODS

### Bioinformatics.

UpsA homologs from several different species were aligned using ClustalW; this alignment was used to create maximum likelihood tree for phylogenetic analysis, as described in the supplemental methods section.

### Culture conditions.

Sulfolobus acidocaldarius strains and derived mutants (Table 1) were grown aerobically at 75°C in basic Brock medium (60), supplemented with 0.1% NZ amine, 0.2% dextrin, and 20 μg/ml uracil, and adjusted to pH 3.5 with sulfuric acid. For solid media, the medium was supplemented with 1.5% gelrite. Plates were incubated for 5 to 6 days at 75°C. Sulfolobus tokodaii strain 7 was grown aerobically at 75°C in basic Brock medium (60), supplemented with 0.1% NZ amine and 0.4% dextrin, and adjusted to pH 3.5 with sulfuric acid. E. coli competent cells DH5α and ER1821 (New England BioLabs [NEB]) used for cloning and methylation, respectively, of plasmid DNA were grown in LB medium (10 g/liter tryptone, 5 g/liter yeast extract, and 10 g/liter NaCl) at 37°C supplemented with the appropriate antibiotics. The growth of cells was monitored by optical density measurements at 600 nm.

### qPCR.

To test the effect of sugars on the transcription of *upsA*, we isolated RNA from cells with or without the addition of mannose (both with and without UV induction). cDNA was synthesized and qPCR performed as described in the supplemental methods.

### Deletion, exchange, and complementation of genes in S. acidocaldarius.

To construct deletion and pilin exchange mutants, up- and downstream flanking regions of the genes of interest (approximately 600 bp) were amplified with primers listed in Table S1. Overlap PCR was performed to connect the up- and downstream fragments. To replace (parts of) *upsA* and *upsB* from S. acidocaldarius with their homologs from Sulfolobus tokodaii, synthetic DNA was ordered (GenScript) consisting of S. acidocaldarius *upsAB* flanking regions and (parts of) *S. tokodaii upsAB* genes (Table S1). The PCR product and synthetic DNA fragments were subsequently cloned into pSVA406, resulting in the plasmids listed in Table S1. All plasmids contain a *pyrEF* cassette allowing selection on plates without uracil. The plasmids were methylated in E. coli ER1821 containing pM.EsaBC4I (NEB) (61) and transformed into S. acidocaldarius MW501 (Δ*fla/*Δ*aap*) (Table 1) (37). This uracil auxotrophic background strain lacks Aap pili and archaella, allowing easy EM analysis. Integrants were selected on plates lacking uracil and grown in 24-well plates for 2 days in the same medium. Subsequently, cultures were plated and grown for 5 days on second selection plates containing uracil and 100 μg/ml 5-fluoroorotic acid (FOA) to select for clones in which the plasmid looped out by homologous recombination. Obtained colonies were tested by PCR for successful deletion/replacement of the genes. Correctness of strains was confirmed by DNA sequencing. Strains that were made during this study are listed in Table 1.

10.1128/mBio.03014-19.10 TABLE S1 Plasmids and primers used during this study. Download Table S1, DOCX file, 0.02 MB. Copyright © 2020 van Wolferen et al. 2020 van Wolferen et al. This content is distributed under the terms of the Creative Commons Attribution 4.0 International license.

For complementation of a Δ*upsAB* mutant (MW143), the DNA region comprising *upsA* and *B* was amplified using primers listed in Table S1 and cloned into pSVA1450 under the control of a maltose-inducible promoter resulting in plasmid pSVA1855 (Table S1). This plasmid was subsequently used as a template to introduce point mutations into *upsA* (D85A, N87A, N94A, and Y96A) (Table 1) using two overlapping primers per mutation (Table S1). Resulting plasmids were then transformed via electroporation into MW143 as described previously (37). Cultures were grown without the addition of uracil. Expression of (mutated) UpsA and B was induced by the addition of 0.2% maltose.

### UV treatment and aggregation assays.

UV light treatment was performed as described in reference 30; a total of 10-ml culture (grown to an optical density at 600 nm [OD_600_], 0.2 to 0.3) was treated with a UV dose of 75 J/m^2^ (254 nm; UV cross-linker; Spectroline) in a plastic petri dish. For FISH experiments, S. acidocaldarius and *S. tokodaii* were first mixed in equal amounts. For complementation the Δ*upsAB* strain, expression of UpsAB (derivatives) was additionally induced with 0.2% maltose. For sugar assays 20 mM mannose, glucose, or *N*-acetylglucosamine was added after UV induction. Afterward, cultures were put back at 75°C for 3 h. Samples taken at different time points were analyzed with phase-contrast microscopy. To quantify aggregated cells after induction with UV, 5 μl of cell culture (diluted to OD_600_ of 0.2 by adding in extra medium and swirling the culture) was spotted and dried on a microscope slide covered with a thin layer of 2% agarose in Brock minimal medium. All pipetting steps were done carefully using tips with their points cut off. Cells were visualized with phase-contrast microscopy (Axio Observer.Z1; Zeiss). Total amounts of free and aggregated cells (≥3) were counted for at least three fields per strain using the ImageJ cell counter. Percentages of cells in aggregates were subsequently calculated. Aggregate sizes were calculated by dividing the total amount of aggregated cells with the amount of aggregates.

### Fluorescence *in situ* hybridization.

For FISH experiments, 10 μl of a mixed UV-induced (described above) culture was spotted and dried on a glass slide. To fix the cells, 10 μl of 37% formaldehyde was spotted on top of the cells, and they incubated for 20 min at room temperature. Afterward, formaldehyde was removed and the cells were washed for 10 min with a drop of 1× phosphate-buffered saline (PBS). Glass slides were subsequently dried at room temperature. Cells were permeabilized by incubating the slides 3 min in 50%, 80%, and 96% ethanol, respectively. After drying the slides, 10 μl of hybridization buffer (900 mM NaCl, 20 mM Tris HCl [pH 8.0], and 10% formamid) mixed with 50 ng/μl FISH probes (for S. acidocaldarius and *S. tokodaii*) (Table S1) was spotted on the cells. Slides were incubated in the dark at 46°C for 1.5 h for hybridization. Subsequently, the cells were washed by incubating the slides for 10 min in wash buffer (450 mM NaCl and 20 mM Tris HCl [pH 8.0]) at 48°C. Slides were then dipped in ice cold water and dried. For microscopy, 1× PBS was spotted on the cells and a coverslip was added. Cells were examined using fluorescence microscopy (Axio Observer.Z1; Zeiss).

### Electron microscopy analysis.

Ups pili on S. acidocaldarius cells were visualized with transmission electron microscopy (TEM). For this, 5 μl of culture was taken 2 h after UV induction and directly dropped onto a carbon‐coated copper grid (SF162-3; Plano), without any prior centrifugation steps. After 2 min of incubation, the fluid was taken off with Whatman paper, and subsequently, 5 μl of 2% uranyl acetate was dropped onto the grid for negative staining and incubated for 30 s. The grid was washed once with water and dried. Transmission electron microscopy images were recorded using the Talos L120C (Thermo Scientific) microscope equipped with a 4,000 by 4,000 Ceta CMOS camera. Acceleration voltage was set to 120 kV and magnification to 2.27 Å/pixel.

### S-layer isolation and *N-*glycan analysis.

A cell pellet from a 1.5-liter *S. tokodaii* strain 7 culture with an OD_600_ of about 0.4 was used to isolate S-layer as described previously (39). The cells were resuspended and incubated while shaking for 60 min at 37°C in 30 ml of buffer A (10 mM NaCl, 0.5% sodium lauroylsarcosine, and a small amount of DNase). Samples were centrifuged for 45 min in an Avanti J-26 XP centrifuge (Beckman Coulter) at 21,000 × *g* (rotor JA-25.50), yielding a brownish tan pellet harboring unsolubilized cell debris with a white top layer (the S-layer fraction). After centrifugation, the white layer on top of the pellet was resuspended in 1 ml buffer B (10 mM NaCl and 0.5% SDS) and incubated for 30 min at 37°C; subsequently, it was spun down in a tabletop centrifuge at maximum speed for 10 min. The latter step was repeated until the pellet was completely white (thereby solubilizing and removing most of the remaining lipids and membrane proteins). The purified S-layer fraction was washed several times with water and then stored in water at 4°C. *N-*linked glycans were released from the purified S-layer and analyzed as described in the supplementary methods.

10.1128/mBio.03014-19.9 FIG S8 *N*-linked glycosylation sites identified from SlaB of *S. tokodaii* by LC-MS^2^ analysis (tryptic digestion and semispecific cleavage search using Byonic software). (A) HCD MS^2^ spectra of glycopeptide ^200^GN[SuphoQuinovose_1_Hex_4_HexNAc_2_]QTISLTLK^209^ from SlaB. (B) HCD MS^2^ spectra of glycopeptide ^347^EIETVN[SuphoQuinovose_1_Hex_4_HexNAc_2_]QTVYTL MNEIK^363^ from SlaB. (C) HCD MS^2^ spectra of glycopeptide ^364^SLN[SuphoQuinovose_1_Hex_4_HexNAc_2_]ASISQLSTTL SSTTTEITTLE NDIK^392^ from SlaB. Download FIG S8, TIF file, 1.0 MB. Copyright © 2020 van Wolferen et al. 2020 van Wolferen et al. This content is distributed under the terms of the Creative Commons Attribution 4.0 International license.
