# Supplementary figures and images for: Species-Specific Recognition of Sulfolobales Mediated by UV-Inducible Pili and S-Layer Glycosylation Patterns
Source: mBio. 2020 Mar 10;11(2):e03014-19. doi: 10.1128/mBio.03014-19 (PMC7064770; doi:10.1128/mBio.03014-19)

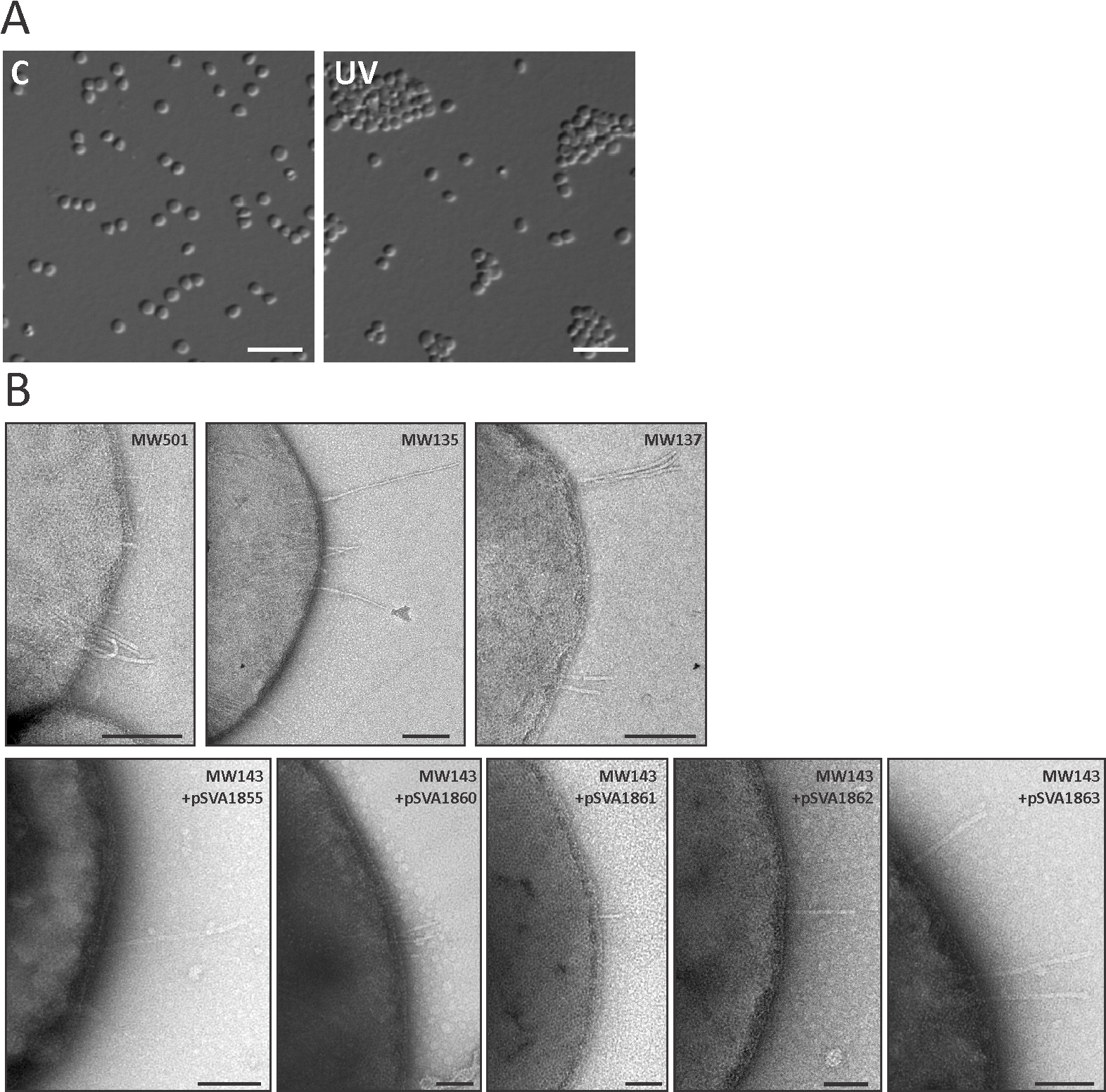

Supplement: FIG S1 [file mBio.03014-19-sf001.tif]

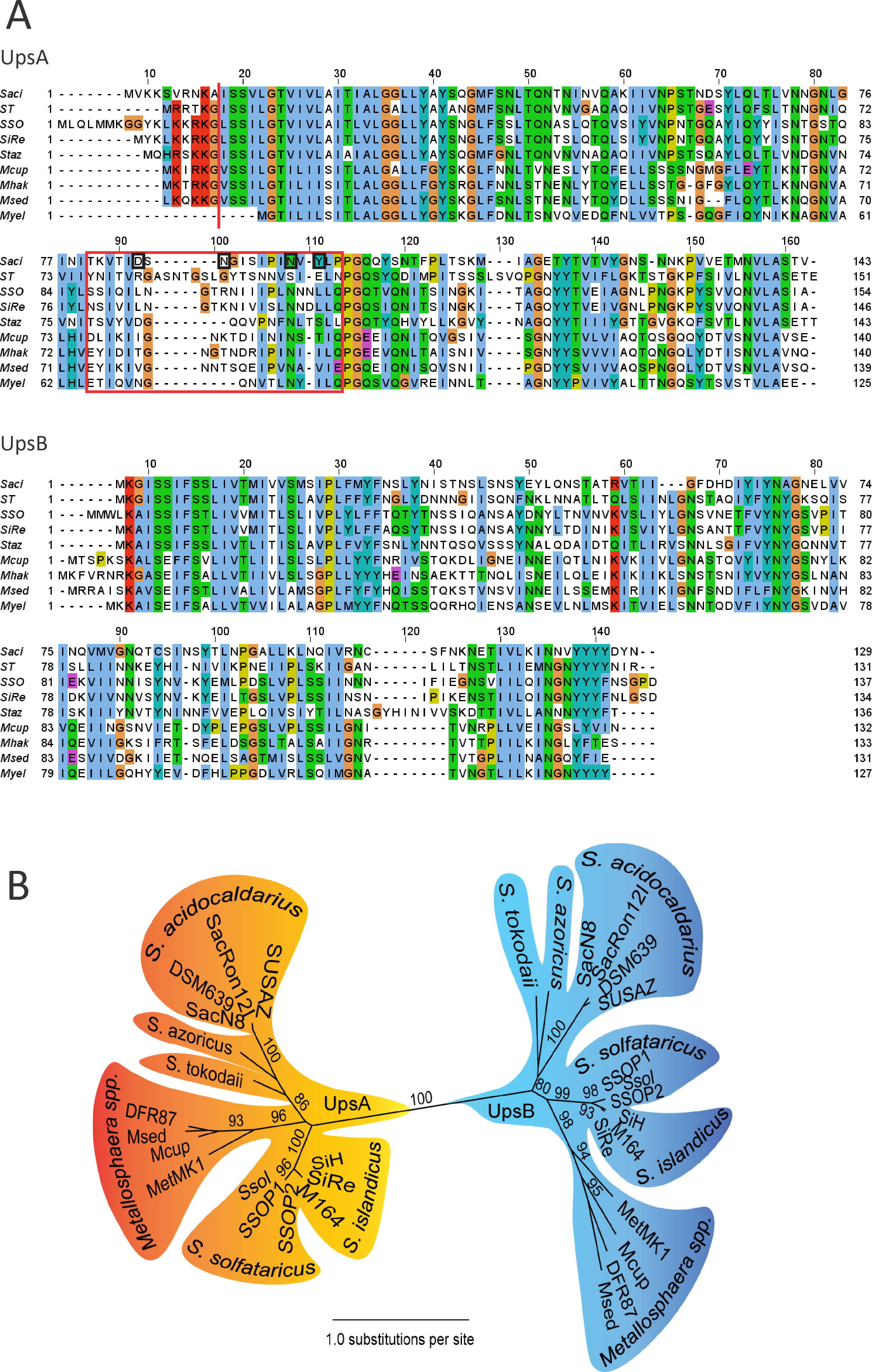

Supplement: FIG S2 [file mBio.03014-19-sf002.tif]

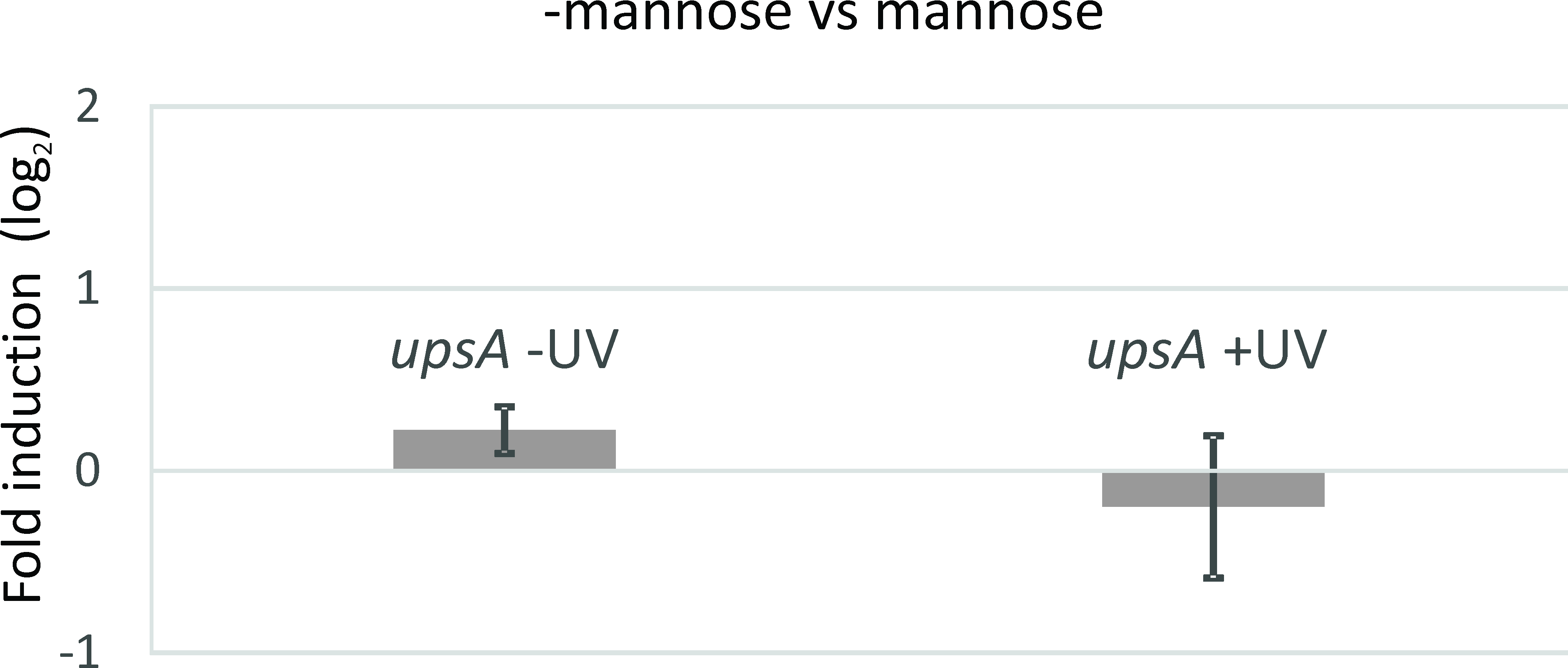

Supplement: FIG S3 [file mBio.03014-19-sf003.tif]

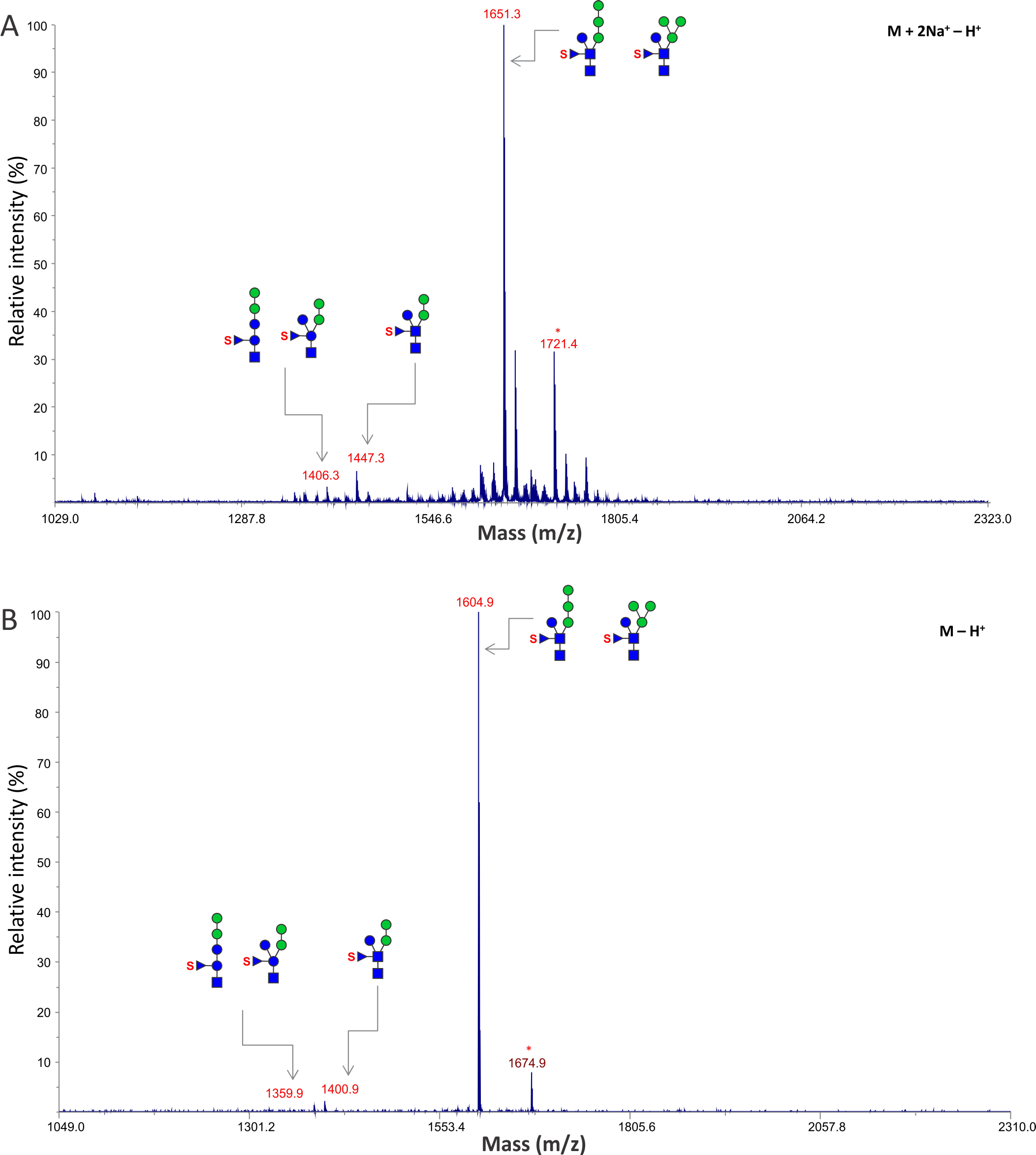

Supplement: FIG S4 [file mBio.03014-19-sf004.tif]

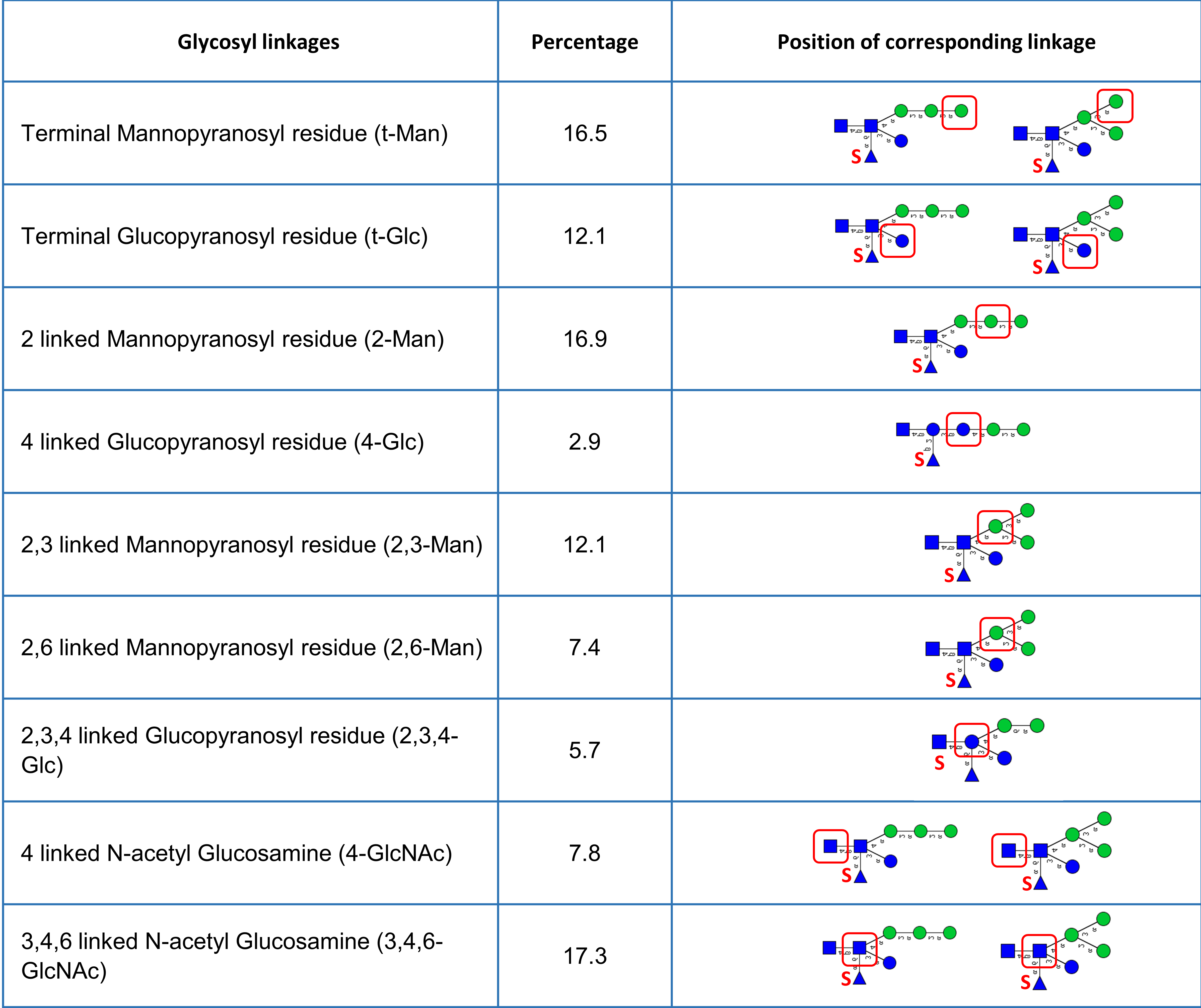

Supplement: FIG S5 [file mBio.03014-19-sf005.tif]

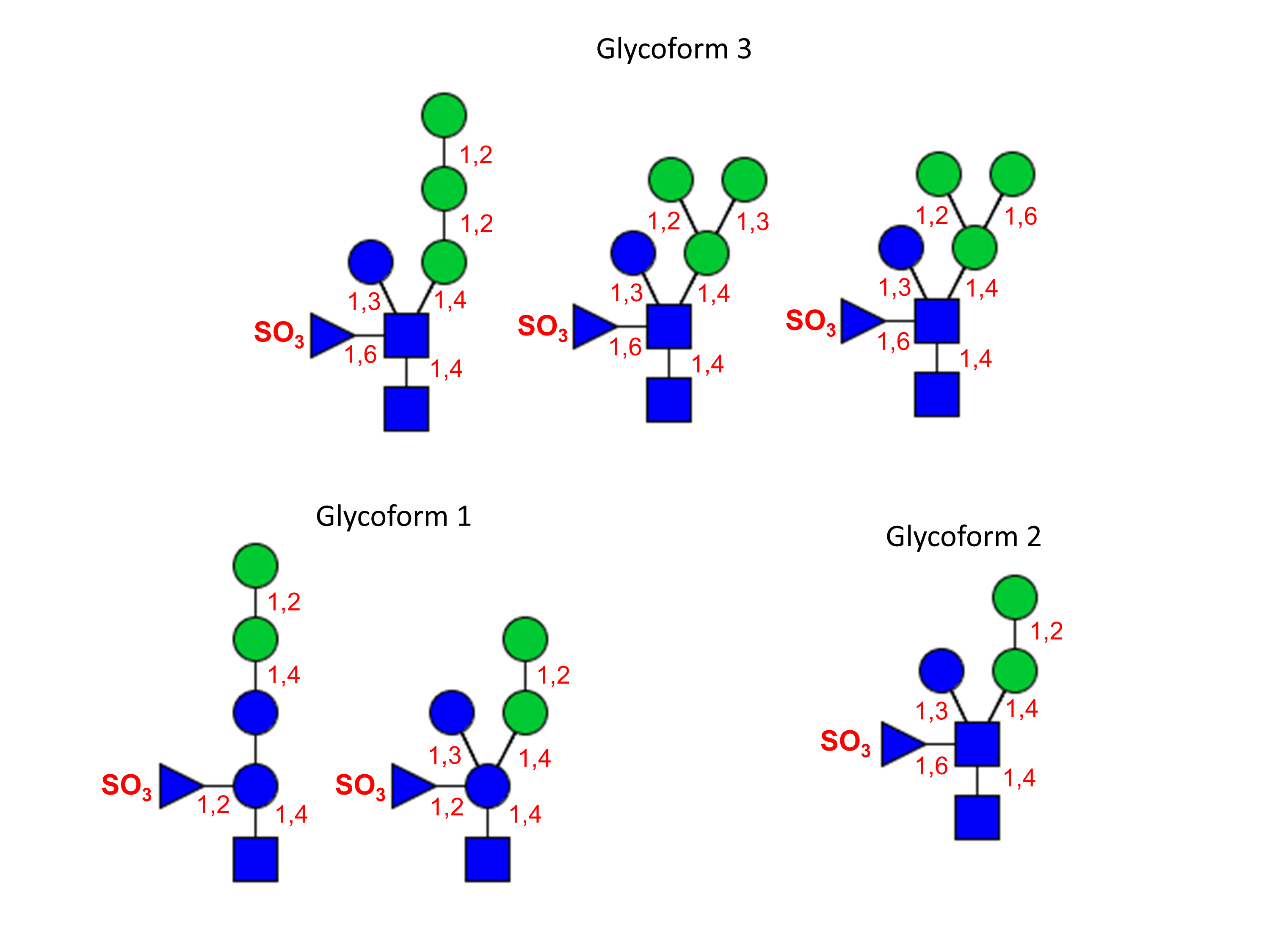

Supplement: FIG S6 [file mBio.03014-19-sf006.tif]

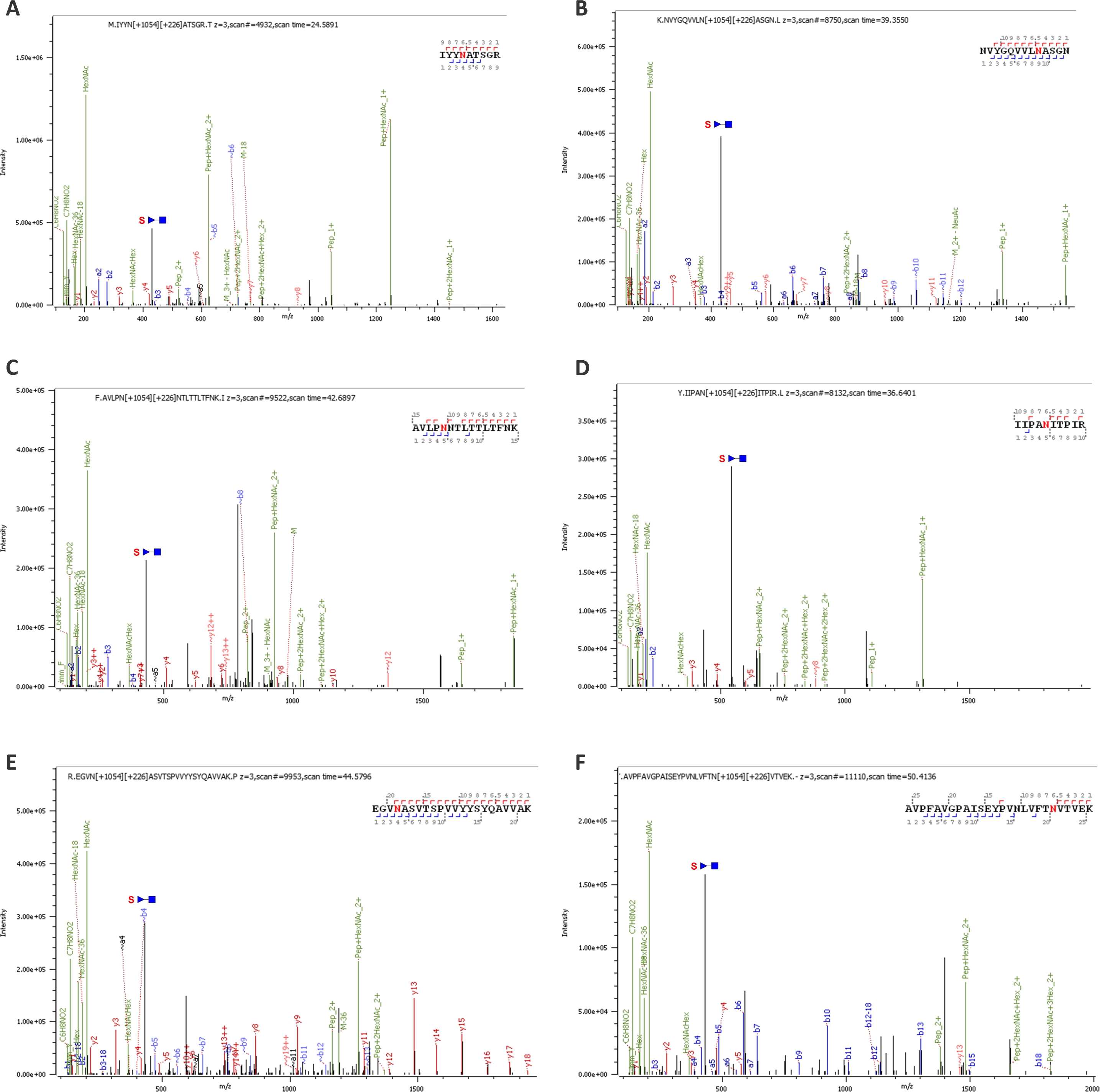

Supplement: FIG S7 [file mBio.03014-19-sf007.tif]

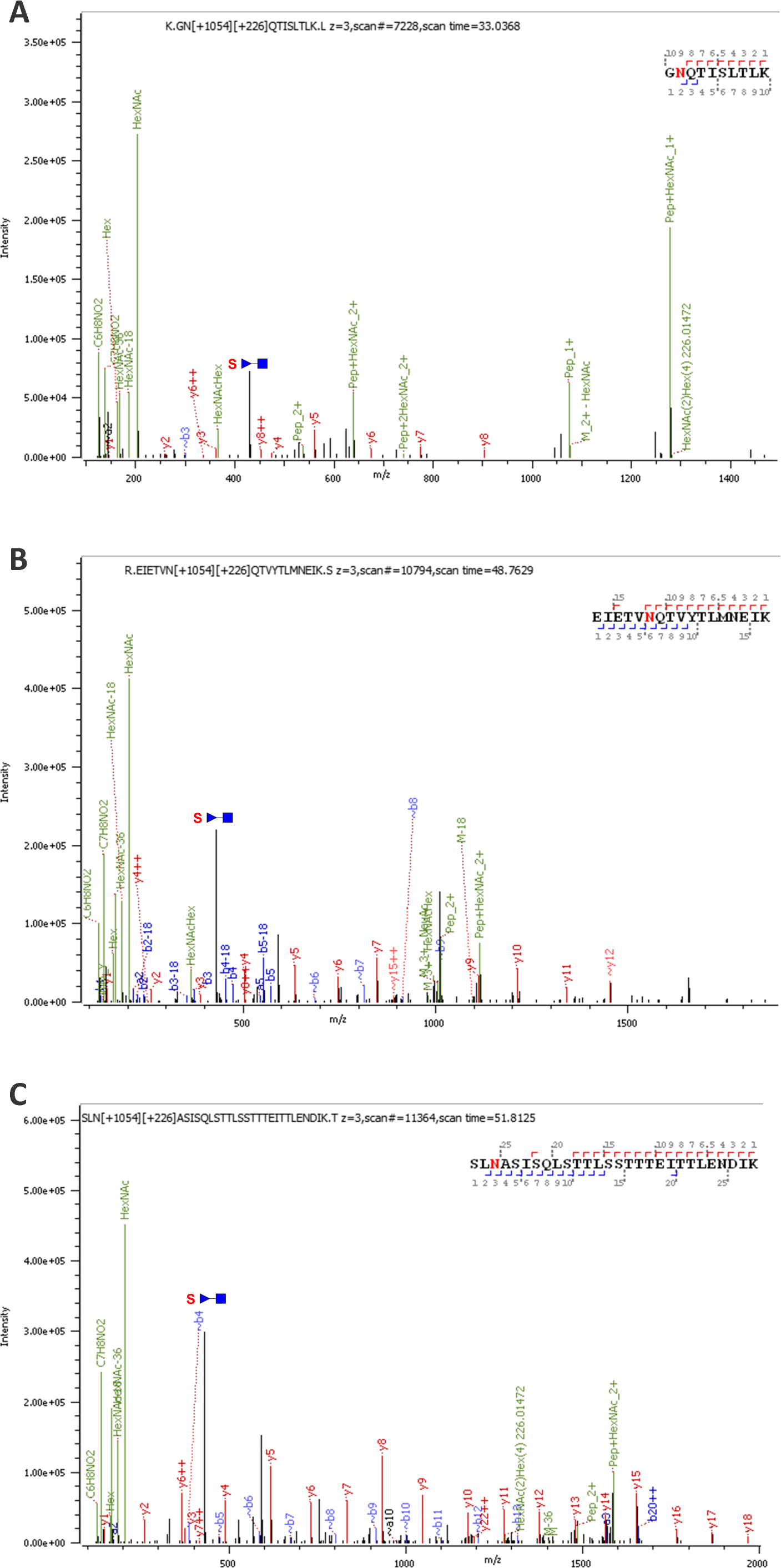

Supplement: FIG S8 [file mBio.03014-19-sf008.tif]
