# Supplementary material for: Species-Specific Recognition of Sulfolobales Mediated by UV-Inducible Pili and S-Layer Glycosylation Patterns
Source: mBio. 2020 Mar 10;11(2):e03014-19. doi: 10.1128/mBio.03014-19 (PMC7064770; doi:10.1128/mBio.03014-19)
Supplement: TEXT S1 [file mBio.03014-19-s0001.docx]

Species-specific recognition of Sulfolobales mediated by UV-inducible pili and S-layer glycosylation patterns

Marleen van Wolferen^1^, Asif Shajahan^2^, Kristina Heinrich^1^, Susanne Brenzinger^3^, Ian M. Black^2^, Alexander Wagner^1^, Ariane Briegel^3,^ Parastoo Azadi^2^, and Sonja-Verena Albers^1^*

**Supplementary results:**

**Phylogenetic analysis of UpsA and UpsB**

In order to study the relationship between UpsA and UpsB homologs, we generated a maximum-likelihood phylogeny on the pilin subunits (Figure S2B). The topology of the tree shows that UpsA and UpsB form distinct clades, suggesting that both pilin subunits are not the result of gene duplication event. It is also evident that UpsA or UpsB homologs from different species are diverging, however, homologs of subspecies are highly similar (>78% identity) or even identical.

**Identification of *N-*linked glycosylation sites of SlaA and SlaB**

To determine different *N-*glycosylation sites in the two S-layer proteins SlaA and SlaB, we specifically searched for glycopeptides containing an oxonium ion with m/z 430 in the HCD MS^2^ spectra, which is a characteristic fragment ion for sulfoquinovose-GlcNAc (Figure S7 and S8). Six glycopeptides from SlaA (Figure S7) and three glycopeptides from SlaB (Figure S8) were detected by our prelimary LC-MS profiling of the tryptic digest. The sequence of each peptide detected was confirmed based on the fragment pattern on the MS^2^ spectra. SlaA has 45 and SlaB has 14 predicted *N*-glycosylation sites, respectively. Further analysis will have to be performed to confirm each of their presence.

**Supplementary methods:**

**Sequence analysis**

To find homologs of UpsA (Saci_1496) and UpsB (Saci_1496b) in other archaeal species, BLAST analysis was performed (1). In order to understand the evolutionary relationship between UpsA and UpsB homologs, they were aligned using ClustalW implemented in Geneious v7.1.7 with a gap opening penalty of 10 and gap extension penalty of 0.1 using a BLOSOM matrix (2). The alignment was manually curated to remove largely gapped or non-homologous sequences. The curated alignment was used to conduct maximum likelihood trees using PHyML (3), implemented in Geneious v7.1.7, with the JTT protein substitution model (4) and 100 bootstraps.

**Accession (numbers from UniprotKB)**

| **Species** | **UpsA homologs** | **UpsB homologs** |
| --- | --- | --- |
| *Sulfolobus acidocaldarius* DSM 639 | Q4J8R3 | Not annotated |
| *Sulfolobus acidocaldarius* N8 | M1IW57 | M1IRR9 |
| *Sulfolobus acidocaldarius* Ron12/I | M1IDL6 | M1JDD3 |
| *Sulfolobus acidocaldarius* SUSAZ | V9S9H2 | V9SBX9 |
| *Sulfolobus solfataricus* P2 | Q7LXX8 | Q7LXX9 |
| *Sulfolobus solfataricus* 98/2 | D0KRG7﻿ | D0KRG6 |
| *Sulfolobus solfataricus* P1 | P95943 | P95944 |
| *Sulfolobus islandicus* REY15A | F0NI59 | F0NIL3 |
| *Sulfolobus islandicus* HVE10/4 | F0NQ96 | F0NKW3 |
| *Sulfolobus islandicus* M.16.4 | C4KJ53 | C4KJ54 |
| *Sulfolobus tokodaii* Str. 7 | Q971F4 | Q971F3 |
| *Stygiolobus azoricus* | Manual | Manual |
| *Metallosphaera sedula* DSM_5348 | not annotated | A4YIJ4 |
| *Metallosphaera cuprina* Ar-4 | F4FYH1 | F4FYH0 |
| *Metallosphaera hakonensis* DSM 7519 | A0A2U9IU74 | A0A2U9IU80 |
| *Metallosphaera yellowstonensis* MK1 | H2C8W2 | H2C8W1 |

**qPCR**

To test the effect of mannose on *upsA* expression (with/without UV induction), RNA was isolated from 10 ml culture 3 hours after UV induction (in biological triplicates), using TRI Reagent (Sigma-Aldrich). DNA was subsequently degraded by incubating the RNA with DNAseI (RNAse free, Fermentas) according to the manufacturer’s protocol. cDNA synthesis was performed using random primers on 1 µg of RNA with the First strand cDNA Synthesis Kit (Roche). qPCR was performed (technical triplicates) in a Rotor-gene Q (Qiagen) qPCR machine using primers 2079 and 2080 (Table S1) with Sygreen Mix Lo-ROX (PCR Biosystems) according to the manufacturers protocol. As a control, primers for housekeeping gene *secY* were used (1480 and 1481, Table S1). ΔΔCt values were calculated to compare transcript levels of *upsA* with or without the addition of mannose. Differences in expression were displayed as log_2_ folds.

**Glycan analysis**

For release of *N-*linked glycans about 100 µg of purified *S. tokodaii*, S-layer was treated with anhydrous hydrazine and incubated at 85°C for 16 hours, a condition that customarily removes *N*-glycans from the sample (5). Hydrazine was then removed by drying with N_2_ gas. A solution of acetic anhydride in sodium bicarbonate was added to the dried sample and incubated 40 minutes for *N-*acetylation followed by mild acid hydrolysis of the sample by Cu (II) acetate. The reaction mixture was passed through a Dowex (H^+^) form for desalting and passed through a C18 sep pak cartridge. The glycans were eluted with 5% acetic acid and dried.

The *N-*linked glycans were permethylated for structural characterization by mass spectrometry (6). For that, the dried eluate was dissolved with dimethyl sulfoxide and methylated by using methyl iodide on a DMSO/NaOH mixture. The reaction was quenched with water and the reaction mixture was passed through a C18 sep pak cartridge. After washing with 5% acetic acid, per-O-methylated sulfated carbohydrates were eluted by isopropanol and dried under a stream of N_2_.

The permethylated glycans were then dissolved with methanol and crystallized with α-dihyroxybenzoic acid (DHBA, 20 mg/mL in 50% v/v methanol:water) matrix. Analysis of the glycans present in the samples was performed in the positive ion mode by MALDI-TOF/TOF-MS using AB SCIEX TOF/TOF 5800 (Applied Biosystem MDS Analytical Technologies).

Permethylated glycans from the samples were infused on an Orbitrap fusion instrument through an ESI probe. The MS^1^ and MS^2^ spectra (HCD) of the glycans were acquired at high resolution by a simple precursor scan and total ion monitoring (TIM) program respectively. Assignment of glycan structures were done manually and by using glycoworkbench software (7), based on the fragmentation patterns.

For glycosyl linkage analysis, the samples were permethylated, depolymerized, reduced, and acetylated; and the resultant partially methylated alditol acetates (PMAAs) analyzed by gas chromatography-mass spectrometry (GC-MS) using a modified version of the previously described procedure (8). The *N*-glycans released from the S-layer proteins were permethylated as mentioned above and the permethylated material was hydrolyzed using 2M TFA (2h in sealed tube at 121 °C), reduced with NaBD^4^, and acetylated using acetic anhydride/pyridine. The resulting PMAAs were analyzed on an Agilent 7890A GC interfaced to a 5975C MSD (mass selective detector, electron impact ionization mode); separation was performed on a 30 m Supelco Equity-1 bonded phase fused silica capillary column.

To create (glyco)peptides, 25 µL of digestion buffer (50 mM aq. NH_4_CO_3_) was added to 20 µL (30.0 µg) of sample protein solution. The protein was reduced, carbamidomethylated and dialyzed against ddH_2_O. The protein sample was digested by adding 5 µL sequencing-grade trypsin (Promega, 0.5 µg/µL) and incubated at 37 ºC for 12h. The digests were desalted by C18 centrifuge cartridges. The digests in elution buffer (80% acetonitrile and 0.1% formic acid) were dried under speed vac. The peptides and glyco-peptides were subsequently re-dissolved in solvent A (0.1% formic acid in water) and stored at -30 ºC until analysis by nano-LC-MS/MS.

Desalted peptides were analyzed on an Orbitrap Fusion instrument (Thermo Scientific) equipped with a nanospray ion source with CID, HCD and ETD fragmentation options and connected to a Dionex binary solvent system. Pre-packed nano-LC columns of 15 cm length with 75 µm internal diameter (id), filled with 2 µm C18 material (reverse phase) were used for chromatographic separation of samples. After the precursor ion scan at 120000 resolution in Orbitrap analyzer, precursors at a time frame of 3 sec were selected for subsequent fragmentation using HCD at normalized collision energy of 28. Another acquisition with a program HCD product triggered ETD, where ETD fragmentation occurs based on the presence of glycan oxonium ions in the HCD fragmentation of the same peptide, was also employed. The threshold for triggering an MS/MS event on ion-trap was set to 500 counts. Charge state screening was enabled, and precursors with unknown charge state or a charge state of +1 were excluded (positive ion mode). Dynamic exclusion was enabled (exclusion duration of 60 s). The fragment ions were analyzed on orbitrap for HCD at 30000 resolution.

The LC-MS/MS spectra of enzymatic digest of S-layer glycoprotein from *S. tokodaii* were analyzed manually with the support of Byonics software. The software search parameter was set as following; Trypsin as digestion enzyme with non-specific cleavage option enabled. Carbamidomethylation as fixed modification, oxidation of methionine and glycan structures identified based on the released glycan analysis as variable modification, was used as search parameters. The HCD MS^2^ spectra of glycopeptides were evaluated for the glycan neutral loss pattern, oxonium ions and the glycopeptide fragmentations to assign the sequence and the presence of glycans in the glycopeptides.

**Supplementary References**

1. Altschul SF, Gish W, Miller W, Myers EW, Lipman DJ. 1990. Basic local alignment search tool. J Mol Biol 215:403–10.

2. Henikoff S, Henikoff JG. 1992. Amino acid substitution matrices from protein blocks. Proc Natl Acad Sci 89:10915–10919.

3. Guindon S, Gascuel O. 2003. A Simple, Fast, and Accurate Algorithm to Estimate Large Phylogenies by Maximum Likelihood. Syst Biol 52:696–704.

4. Jones DT, Taylor WR, Thornton JM. 1992. The rapid generation of mutation data matrices from protein sequences. Comput Appl Biosci 8:275–82.

5. Patel T, Bruce J, Merry A, Bigge C, Wormald M, Jaques A, Parekh R. 1993. Use of hydrazine to release in intact and unreduced form both N- and O-linked oligosaccharides from glycoproteins. Biochemistry 32:679–93.

6. Anumula KR, Taylor PB. 1992. A comprehensive procedure for preparation of partially methylated alditol acetates from glycoprotein carbohydrates. Anal Biochem 203:101–8.

7. Ceroni A, Maass K, Geyer H, Geyer R, Dell A, Haslam SM. 2008. GlycoWorkbench: a tool for the computer-assisted annotation of mass spectra of glycans. J Proteome Res 7:1650–9.

8. Heiss C, Wang Z, Black I, Azadi P, Fichorova RN, Singh BN. 2016. Novel structural features of the immunocompetent ceramide phospho-inositol glycan core from Trichomonas vaginalis. Carbohydr Res 419:51–9.
