# Supplementary material for: Species-Specific Recognition of Sulfolobales Mediated by UV-Inducible Pili and S-Layer Glycosylation Patterns
Source: mBio. 2020 Mar 10;11(2):e03014-19. doi: 10.1128/mBio.03014-19 (PMC7064770; doi:10.1128/mBio.03014-19)
Supplement: TABLE S1 [file mBio.03014-19-st001.docx]

**Table S1:** Plasmids and primers used during this study.

| Plasmids | | Backbone | Description | Source/reference |
| --- | --- | --- | --- | --- |
|  | pSVA406 |  | Backbone of deletion and exchange plasmids. | (Wagner *et al.*, 2012) |
|  | pSVA1450 |  | Backbone for expression plasmids containing a maltose inducible promoter. | (Wagner *et al.*, 2014) |
|  | pSVA1803 | pSVA406 | Deletion plasmid for *upsAB (saci1496+saci1496b)* | This study |
|  | pSVA1820 | pSVA406 | To exchange *Saci upsAB* with *ST upsAB* | This study |
|  | pSVA1822 | pUC57 | Part UpsA Saci replaced by same part in *S tokodaii* synthetic | Ordered from Genscript |
|  | pSVA1824 | pSVA406 | To exchange *Saci upsA* (aa 84-98) with ST *upsA* (aa 80-101) | This study |
|  | pSVA1855 | pSVA1450 | P_mal_ upsA+upsB (*upsA* without first 4 bp) | This study |
|  | pSVA1860 | pSVA1855 | P_mal_ upsA+upsB (*upsA* without first 4 bp); D85A pointmutation in *upsA* | This study |
|  | pSVA1861 | pSVA1855 | P_mal_ upsA+upsB (*upsA* without first 4 bp); N87A pointmutation in *upsA* | This study |
|  | pSVA1862 | pSVA1855 | P_mal_ upsA+upsB (*upsA* without first 4 bp); N94A pointmutation in *upsA* | This study |
|  | pSVA1863 | pSVA1855 | P_mal_ upsA+upsB (*upsA* without first 4 bp); Y96A pointmutation in upsA | This study |
|  | |  |  |  |
| Primers /  probes | | **Description** | **Sequence** | **Purpose** |
|  | **Saci_FISH** |  | [AF488}ATAGGTTCGGTGGGCCTT | *S. acidocaldarius* FISH-probe, label: AF488 |
|  | **Stok_FISH** |  | [TxRd]ATAGGTTTGGCGGGCCG | *S. tokodaii* FISH-probe, label: TexasRed |
|  | 1480 | qPCR *secY* fw | CCTGCAACATCTATCCATAACATACCGA | qPCR on *secY* |
|  | 1481 | qPCR *secY* rv | CCTCATAGTGTATATGCTTTAGTAGTAG | qPCR on *secY* |
|  | 2000 | *ΔupsA*_US_FW_ApaI | GTAGGGCCCCCAGTTAGTTAAGCTTTTACCAG | To create pSVA1803 and pSVA1820 |
|  | 2007 | *ΔupsB*_DS_RV_BamHI | GCGGATCCAAACCACATCAGCTGTCTTATCAC | To create pSVA1803 |
|  | 2008 | *ΔpsAB*_US_RW | GAATAGAATAGTTTTAACCAACTTTCCTCAAATAAAATG | To create pSVA1803 |
|  | 2009 | *ΔpsAB*_DS_FW | GAAAGTTGGTTAAAACTATTCTATTCTTTTTTAG | To create pSVA1803 and pSVA1820 |
|  | 2079 | qPCR *upsA* fw | TAGCCAGGGTATGTTCAGTAATC | qPCR on *upsA* |
|  | 2080 | qPCR *upsA* rv | ACCTAAGTTCCCGTTATTGAC | qPCR on *upsA* |
|  | 3001 | *upsAB* Stok_US_rv | CTTCTCATATTGTTCCCCATCTTTCCTCAAATAAAATGAATC | To create pSVA1820 |
|  | 3002 | *upsAB* Stok_gene_fw | GAGGAAAGATGGGGAACAATATGAGAAG | To create pSVA1820 |
|  | 3003 | *upsAB* Stok_gene_RV | GAATAGAATAGTTTATCTAATATTATAGTAATAATAATTACC | To create pSVA1820 |
|  | 3004 | *upsAB* Stok_DS_FW | CTATAATATTAGATAAACTATTCTATTCTTTTTTAG | To create pSVA1820 |
|  | 3065 | *upsB*_rv_EagI | CACGCGGCCGTCAATTGTAATCATAGTAATAG | To create pSVA1855 |
|  | 3066 | *upsA*_fw_NcoI | CGCTCCATGGTTAAAAAATCCGTAAGAAAT | To create pSVA1855 |
|  | 3067 | UpsA_fw_D85A | AAGTAACTATAGCTTCTAATGG GATATCGATCC | To create pSVA1860 |
|  | 3068 | UpsA_rv_D85A | CCATTAGAAGCTATAGTTACTTTCGTTATATTTATACC | To create pSVA1860 |
|  | 3069 | UpsA_fw_N87A | TATAGATTCTGCTGGGAT ATCGATCCCAATA | To create pSVA1861 |
|  | 3070 | UpsA_rv_N87A | ATCCCAGCAGAATCTATAGTTACTTTCGTTATATTTATAC | To create pSVA1861 |
|  | 3071 | UpsA_fw_N94A | CCAATAGCTGTGTATTTACCTCCTGGTC | To create pSVA1862 |
|  | 3072 | UpsA_rv_N94A | GTAAATACACAGCTATTGGGATCGATATCCCAT | To create pSVA1862 |
|  | 3073 | UpsA_fw_Y96A | AATAAATGTGGCTTTACCTCCTGGTCAGCAAT | To create pSVA1863 |
|  | 3074 | UpsA_rv_Y96A | GGTAAAGCCACATTTATTGGGATCGATATC | To create pSVA1863 |
